# A New Species of the Genus *Gekko* (Squamata: Sauria: Gekkonidae) from the Dabie Mountains, China †

**DOI:** 10.3390/ani13243796

**Published:** 2023-12-08

**Authors:** Caiwen Zhang, Afang Wu, Bo Cai, Lanrong Wang, Dapeng Pang, Haohao Ma, Lei Yu, Xiangyang Li, Hua Huang, Lin Zeng, Li Li, Jie Yan, Peng Li, Baowei Zhang

**Affiliations:** 1 School of Life Sciences, Anhui University, Hefei 230601, China; d23101003@stu.ahu.edu.cn (C.Z.); af631499742@163.com (A.W.); d22201015@stu.ahu.edu.cn (L.W.); pia@163.com (D.P.); 18715134863@163.com (H.M.); yulei2692@163.com (L.Y.); 2 Chengdu Institute of Biology, Chinese Academy of Sciences, Chengdu 610299, China; caibo@cib.ac.cn; 3 Liankang Mountains National Nature Reserve Affairs Center, Xinyang 465550, China; xiangyanglee@126.com; 4 Luoshan County Dongzhai National Nature Reserve Affairs Center, Xinyang 465322, China; zwp7054@163.com; 5 Henan Dabie Mountains National Nature Reserve Administration, Xinyang 464236, China; 13937667106@139.com (L.Z.); lili0215313@163.com (L.L.); 6 College of Life Sciences, Nanjing Normal University, Nanjing 210023, China; yanjie@njnu.edu.cn

**Keywords:** *Gekko kaiyai* **sp. nov.**, Gekkonidae, Central China, molecular phylogenetic analysis, morphology

## Abstract

**Simple Summary:**

The genus *Gekko* Laurenti, 1768, currently comprises approximately 86 species, of which 21 are native to China, that commonly live on walls, rocks, and trees, and are distributed across Southeast Asia, western Oceania, and Melanesia. This article describes a new species of *Gekko* (Squamata: Gekkonidae) based on its distinct morphological features and molecular evidence, which was identified in the Dabie Mountains on the border of Anhui and Henan provinces of Central China. The analysis of phylogeny based on a mitochondrial DNA fragment (16S, CYTB, and COI) indicated that the new taxon is different from its congeners. Morphologically, the new species can be diagnosed from the other subgenus *Japonigekko* species by a combination of 34 (14 mensural and 20 meristic) morphological characteristics, and Principal component analysis (PCA) and one-way analysis of variance (ANOVA) showed that the new species can be clearly distinguished from its sister species *G. hokouensis*. Based on the above multiple lines of evidence, we describe this gecko from the Dabie Mountains as a new species, *Gekko kaiyai* **sp. nov.** The discovery of this species implies that there are now 87 identified species in the genus *Gekko*, 22 of which can be found within China.

**Abstract:**

This study describes a novel species of *Gekko* (Squamata: Gekkonidae) based on its distinct morphological features and molecular evidence, which was identified in the Dabie Mountains on the border of Anhui and Henan provinces of Central China. *Gekko kaiyai* **sp. nov.** could be distinguished from its congeners owing to its morphological characteristics, such as being a medium body sized gecko species (snout–vent length, 56.98–64.99 mm, n = 4, females; 50.03–61.56 mm, n = 11, males); nostrils scale in contact with rostral scale; tubercles on the dorsal and limb, while the upper forelimb is smooth with no tubercles; 22–33 interorbital scales between the anterior corners of the eyes; 157–209 ventral scales between mental and cloacal slit; 90–121 midbody scale rows; 30–43 ventral scale rows; 7–9 sub-digital lamellae on first fingers, 8–13 fourth fingers, 7–9 first toes, and 7–11 fourth toes; free of webbing in the fingers and toes; 9–12 pre-cloacal pores in males, which are absent in females; post-cloacal unilateral tubercles 1 (few 2); and a dorsum that is greyish white to dark brown, with 6–7 brown markings between the nape and sacrum. The phylogenetic tree based on the mitochondrial DNA sequences (16S, CYTB, and COI) indicated that *Gekko kaiyai* **sp. nov.** form an independent clade with strong support (100/1) and are a sister group to *G. hokouensis*. At the inter-species level, the genetic distances were all large, further confirming that an independent species had been identified. The discovery of this species implies that there are now 87 identified species in the genus *Gekko*, 22 of which can be found within China.

## 1. Introduction

The genus *Gekko* Laurenti, 1768, refers to nocturnal reptiles that commonly live on walls, rocks, and trees, and are distributed across Southeast Asia, Western Oceania, and Melanesia [1,2]. The genus, *Gekko*, currently comprises approximately 86 species [3], of which 21 are native to China [4,5,6]. The most recent phylogenetic analysis and taxonomic revision divided the *Gekko* members into seven subgenera [1]: *Archipelagekko* (Taylor, 1919), *Balawangekko* (Brown, 2010)), *Gekko* (Stejineger, 1936), *Japonigekko* (Dumeriland Bibron, 1836), *Lomatodactylus* (Vander Hoeven, 1833), *Ptychozoon* (Stejineger, 1902), and *Rhacogekko* (Boulenger, 1899). The subgenus, *Japonigekko*, is the most ecologically and morphologically diverse group in the genus and currently includes 31 species worldwide [3]. This subgenus is widely distributed in East Asia and specifically east of China and Japan, south of Vietnam and Laos [1,4,7]. Members of the subgenera *Japonigekko* can be identified by their morphological characteristics, which include a relatively moderate size, 2–3 nasals, nares usually in contact with rostral scale, possession or lack of dorsal tubercle rows (0–21), possession of up to 32 pre-cloacal pores (0–32), and lateral folds without tubercles [1].

The Dabie Mountains are located at the junction of the Anhui, Henan, and Hubei Provinces, and this area forms the watershed between the Yangtze and Huai Rivers. It has a warm and humid monsoon climate in the North subtropical zone, and its superior geographical environment and suitable climatic conditions indicate that it is rich in animal and plant resources. According to previous records, two species of the genus *Gekko* (*G. hokouensis* and *G. japonicus*) have been identified in the Dabie Mountains [8]. During herpetological surveys in the Dabie Mountains from August to September 2022, some *Gekko* specimens were collected that were not *G. hokouensis* or *G. japonicus*. Based on molecular phylogenetic analyses and morphological comparisons, a new species was identified as an undescribed taxon of the genus *Gekko*.

## 2. Materials and Methods

### 2.1. Sampling

**Figure 1:**
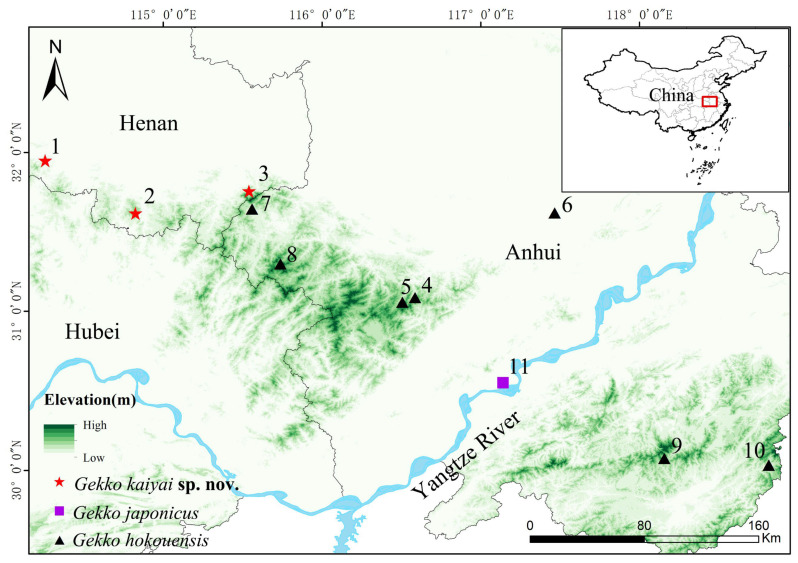
Map of the geographic distribution of *Gekko* (*Japonigekko*) used in this study. Numerals indicate sample sites: (1) Luoshan County, Henan; (2) Xin County, Henan; (3) Shangcheng County, Henan; (4) Lujiang County, Anhui; (5) Shucheng City, Anhui; (6) Hefei City, Anhui; (7) Jinzhai County, Anhui; (8) Jinzhai County, Anhui; (9) Huangshan City, Anhui; (10) Huangshan City, Anhui; (11) Wangjiang County, Anhui.

From August to September 2022, 17 gecko individuals (two subadult, four adult females, and 11 adult males) were collected from the Dabie Mountains in the border region of the Anhui and Henan Provinces, China (Figure 1). For comparison, 25 specimens of *G. hokouensis* and two specimens of *G. japonicus* from the Dabie Mountains and surrounding areas of Anhui Province were also collected. After taking photographs, the individuals were euthanized (using isoflurane) and fixed in 10% formaldehyde for 2 d, and then finally washed and preserved in 75% ethanol. The study received ethical review and approval from the Institutional Animal Care and Use Committee of School of Life Sciences, Anhui University (project number IACUC(AHU)-2022-050). Vouchered specimens for this work were deposited at the Anhui University Biology Museum (AHUBM). 

### 2.2. Molecular Data and Phylogenetic Analyses

Liver tissue samples were taken from all geckos and preserved in 95% ethanol prior to fixation. Purified DNA was then obtained from the liver tissues of individuals using a standard phenol/chloroform extraction method [9,10]. 

Mitochondrial gene segments encoding the 16S ribosomal RNA gene (16S), cytochrome b (CYTB), and cytochrome oxidase subunit I (COI) were selected, and the homologous regions were amplified using the primers previously described by Lyu et al. (2021) [6] and Kumazawa and Endo (2004) [11]. The L3975 (5′-CGCCTGTTTACCAAAAACAT-3′) and H4551 (5′-CCGGTCTGAACTCAGATCACGT-3′), primers were used for 16S, L-14731 (5′-GAAAAACTATCGTTGTTATTCAACTA-3′) and H–Thr-2 (5′-GTTTACAAGGTCAGCGCTTT-3′) primers were used for CYTB [6], and rCOI-1H (5′-TAGTGGAARTGKGCTACTAC-3′) and rTrp-1L (5′-TAAACCARGRGCCTTCAAAG-3′) primers were used for COI [11]. The PCR amplification system volume was 25 μL, and this included 2 μL of template DNA, 1 μL of the upper and lower primers, 12.5 μL of Taq polymerase, and 9.5 μL of ddH2O. The PCR cycle parameters for the 16S and CYTB included an initial denaturation step at 95 °C for 4 min, followed by 35 cycles at 95 °C for 40 s, annealing at 53 °C for 34 s, and then expansion at 72 °C for 1 min; this was followed by a final extension step for 10 min of 72 °C [6]. The COI amplification was performed under the following conditions: 1 min at 94 °C, followed by 5 cycles at 94 °C for 1 min, 1.5 min at 48 °C, and 1.5 min at 72 °C, followed by 35 cycles of 1 min at 94 °C, 1.5 min at 58 °C, and 1.5 min at 72 °C, and then a final 5 min at 72 °C [2]. The PCR amplified products were sent to General Biology (Anhui) Co., Ltd. (Chuzhou, Anhui, China), and the derived sequences were stored in GenBank (for GenBank accession numbers, see Table 1).

According to previous studies, the gene sequences for all species in the subgenus *Japonigekko* from GenBank were downloaded for use in extensive phylogenetic comparisons. Moreover, for phylogenetic analysis, the available sequence data for *Gekko* (*Gekko*) gecko (Linnaeus, 1758) were downloaded and used to aid in outgroup comparisons. Overall, 105 sequences were selected for analyses in this study, and these were sourced from 47 gecko individuals and 16 species. (For all GenBank accession numbers, see Table 1).

The 16S, CYTB, and COI sequences were aligned using MAFFT 7.110 with the G-INI-i option [13,14]. The aligned sequences were then sheared in BioEdit 7.0.5.3 to remove incorrect base sequences from the head and tail region [15]. The datasets for the 16S, CYTB and COI regions were then combined manually. Before multi-gene phylogenetic tree reconstruction, the jModelTest was used to estimate the best-fit evolutionary model for the alignment using the calculation from the corrected Akaike information criterion [16,17]. The results showed that the GTR + I + G model was the best partition. The 16S, CYTB, and COI combined, sequenced datasets were analyzed using Bayesian inference (BI) in MrBayes 3.2.4 [18], and maximum likelihood (ML) was performed using RaxmlGUI 1.3 [19]. Two independent runs were conducted using the BI analysis (each of which was performed for 10,000,000 generations and sampled every 1000 generations with the first 25% of samples discarded as burn-in and the remaining 75% retained), and used to construct a 50% majority consensus tree and Bayesian posterior probabilities (BPPs) were calculated. This process generated a potential scale reduction factor (PSRF) of <0.005, which confirmed that the trees had error rates 0.01. Markov Chain Monte Carlo simulation convergence was assessed using Tracer v1.5 [20], and the results were verified using the ESSs of all parameters that exceeded 200 with PSRFs close to 1000.

### 2.3. Morphological Analyses

In accordance with previous studies, 34 (14 mensural and 20 meristic) commonly -used morphological characteristics were assessed in the adult gecko samples using previously described terminologies and methods [2,5,6]. Morphological measurements were generally taken to the nearest 0.01 mm using digital calipers (DEGUQMNT, 0–150 mm); however, the characteristics with small values (<10 mm) were measured using a digital stereoscopic binocular microscope. The distance from the tip of the snout to the posterior edge of the vent was the snout–vent length (SVL), from the posterior margin of the cloaca to the tip was the tail length (Tal), the minimum distance between the axilla and groin on a straightened body was the axilla to groin distance (AG), the tip of the snout to the posterior margin of the ear opening was the head length (HL), the maximum head width was the head width (HW), the maximum head height was the head height (HH), the distance from the snout tip to anterior corner of the eye was the snout-eye distance (SE), the distance between the posterior margin of the eye and the posterior margin of the ear opening was the eye-ear distance (EE), greatest diameter of orbit (ED), maximum diameter of the ear opening (TD), the maximum rostral scale width was rostral scale width (RW), the maximum rostral scale height was the rostral scale height (RH), the maximum mental width was mental width (MW), and the maximum mental length was indicated as mental length (ML).

Considering that sexual dimorphism may exist within geckos, sexes were separated for subsequent comparisons among the samples. At the same time, to account for the possible influence of allometry, subadults were omitted from the specimen’s data and then scaled to remove allometric effects of body size using the following equation: Xadj = log(X) − β[log(SVL) − log(SVLmean)], where Xadj = adjusted value; X = measured value; β = unstandardized regression coefficient for each population; and SVLmean = overall average SVL of all populations [21,22,23,24]—accessible in the R package GroupStruct (available at https://github.com/chankinonn/GroupStruct, accessed on 11 November 2023). The morphometrics of each species were adjusted separately and then concatenated prior to analysis so as not to conflate intra- with interspecific variation [25,26]. One-way analysis of variance (ANOVA) tests were used to evaluate significant differences in the morphometric characteristics between the different species, with a *p* < 0.05 in the Levene’s test. Using the dplyr package in R to perform Bartlett’s test of sphericity on the original variables, the data were checked as suitable for Principal component analysis (PCA), and then Kaiser–Meyer–Olkin Measure (KMO) of Sampling Adequacy analysis to screen the variables (communality < 0.5). PCA implemented by the factoextra package in R was employed to extract PCA analysis result information and draw PCA plots. All statistical analyses were performed with R version 4.2.2.

The meristic characteristics and their abbreviations were as follows: naso-rostrals, supra-nasals, and post-nasals as nasals (N); scales between supra-nasals, in contact with rostral scale as intersupra-nasals (I); the number of scales from the commissure of the jaw to the rostral scale as supra-labials (SPL); number of scales from the commissure of the jaw to the mental scale as infra-labials (IFL); number of scales in a line between the anterior corners of the eyes as interorbitals (IO); number of scales in a line from the nostril to the anterior corner of the eye as preorbitals (PO); post-mentals (PM); gulars bordering the post-mentals (GP); dorsal tubercle rows at the midbody (DTR); granules surrounding dorsal tubercles (GSDT); scales in a line from the mental to the front of the cloacal slit (SMC); scale rows at the midbody (including ventral scales, SR); ventral scale rows at midbody (V); sub-digital lamellae under entire first finger (LF1); sub-digital lamellae under entire fourth finger (LF4); sub-digital lamellae under entire first toe (LT1); sub-digital lamellae under entire fourth toe (LT4); pre-cloacal pores (PP); and post-cloacal tubercles (PAT). Bilateral scale counts are given as left/right.

Morphological data of the *G. hokouensis* were obtained from specimens in the Dabie Mountains, Anhui Province, and from the literature [27,28]. For other *Japonigekko* species subgenera, morphological data were obtained from the literature [2,5,6,7,27,28,29,30,31,32,33,34,35,36,37,38,39,40,41,42,43,44,45].

## 3. Results

### 3.1. Phylogenetic Analyses

**Figure 2:**
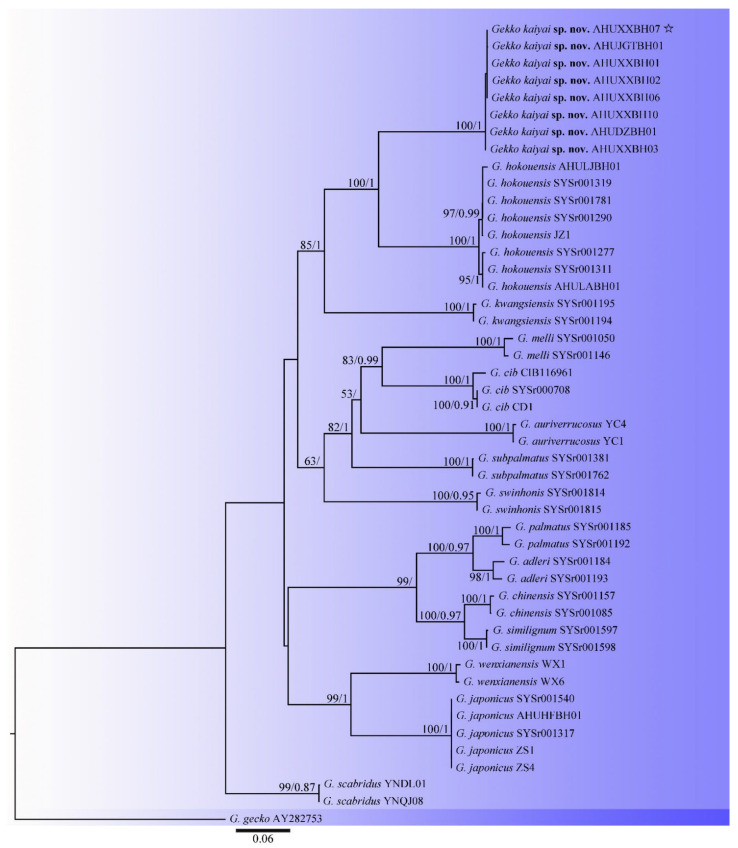
Maximum-likelihood and Bayesian inference phylogenies based on mitochondrial 16S rRNA, CYTB, and COI genes. ML Bootstrap Support (BS)/Bayesian Posterior Probabilities (PP) at nodes. If BS < 50 or PP < 0.90, it will not be displayed; “☆” indicates holotype gene sequences.

The aligned dataset contained data from 46 individuals from the subgenera *Japonigekko* species and one individual from the outgroup *Gekko gecko* species (Table 1). The combined dataset for the 16S, CYTB, and COI regions included 44 collections that represented 16 taxa and resulted in a concatenated alignment of 2474 characters with GTR + I + G as the best-fit evolutionary model. The ML search stopped after 150 BS replicates. For the BI, all chains converged after 10 million generations with an average standard deviation of split frequencies of 0.004379; the average ESS was 5636. The ML and BI algorithms generated similar topologies in the main lineages; thus, only the topology generated by the ML algorithm is presented along with the BS value and BPPs > 50% and 0.90, respectively, at the nodes. Eight specimens for the undescribed species formed an independent clade with strong support (100/1) and were a sister group to *G. hokouensis* (Figure 2).

To further explore the species relationships among the subgenus *Japonigekko*, the alignment with 14 selected CYTB sequences, 12 selected 16S sequences, and 7 selected COI sequences underwent a genetic distance analysis. The uncorrected pairwise divergences within these specimens ranged from 6.8% to 29.4% for the CYTB sequences (Table S1), 2.1% to 19.1% for the 16S sequences (Table S2), and 18.8% to 25.9% for the COI sequences (Table S3). For the CYTB, the genetic distance between the undescribed species and other known species ranged from 18.6% (*G. hokouensis)* to 27.8% (*G. chinensis*). According to the statistical analyses, all genetic distances between species were >14.0% except for those between *G. adleri* and *G. palmatus*, and *G. chinensis* and *G. similignum*, which were 7.9% and 6.8%, respectively. For the 16S rRNA sequences, the genetic distance between the undescribed species and other known species ranged from 9.3% (*G. hokouensis)* to 19.0% (*G. melli*), and comparison analysis showed that the undescribed species and *G. hokouensis* were closely related. Taken together, the genetic distance between the undescribed species and the subgenera *Japonigekko* was found to be high enough to indicate a new species.

### 3.2. Morphological Analyses

The results of the ANOVA indicated that the new taxon group was significantly different from the closely-related species (*G. hokouensis*) in many morphometric characters (*p*-values < 0.05; Table 2); the males’ significant differences were characteristics in AG, HW, HH, ED, TD and MW, whereas females’ were characteristics in SVL, AG, HL, HW, HH, ED, TD, and RW.

**Figure 3:**
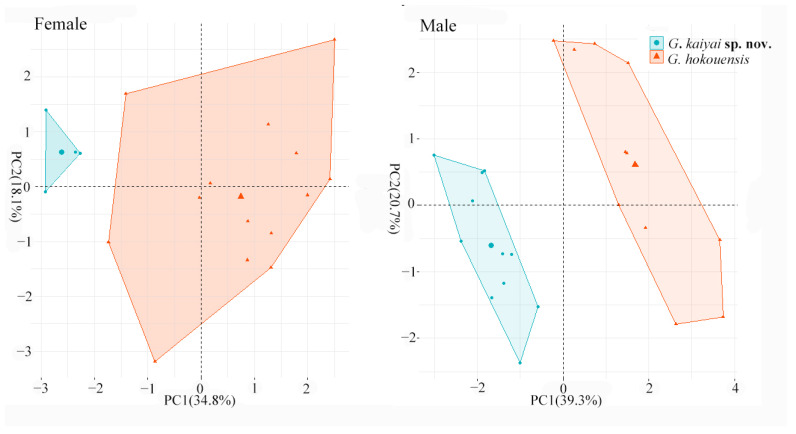
Principal component analysis performed for *Gekko kaiyai* **sp. nov.** and *G. hokouensis* based on 10 commonly used morphological traits (except Tal, SE, RW, ML). Numbers inside the brackets indicates the percentages of the total variance explained by each axis.

The results of the Bartlett’s test of sphericity show that there are correlations among variables (*p* < 0.05) which indicated that PCA can be performed. We found most variables have at least a moderate correlation value (i.e.,: >0.3 or <−0.3), except (SE, RW, ML). So, we omitted the variable with low correlation variables. At the same time, the Kaiser–Meyer–Olkin measure (KMO) of sampling adequacy analysis shows an overall Measures of Sampling Adequacy (MSA) of 0.76 (>0.5). In PCA analysis, the first four principal components explained 79.24% of the total variation in the males, where PC1, PC2, PC3, and PC4 eigenvectors accounted for 39.30%, 20.70%, 9.88%, and 9.35% of the total variance, respectively (Table 3). Similarly, the first four principal components occupied a considerable proportion in the females, 76.89% of the total, whereas the PC1, PC2, PC3, and PC4 eigenvectors accounted for 34.83%, 18.12%, 14.06%, and 9.88% of the total variance, respectively (Table 3). Regardless of the sex of the two aforementioned species, the samples showed intraspecific polymerization; moreover, all samples showed interspecific detachment, as evidenced by the fact that there was no overlap between the two species on the two-dimensional graphs for PC1 and PC2 (Figure 3). The results of the ANOVA and PCA indicated that the unnamed populations were significantly different from the closely-related species.

#### 3.2.1. Taxonomic Accounts

The results of the molecular phylogenetic analyses and morphological comparison all indicated that the new taxon of the genus *Gekko* (*Japonigekko*) from the Dabie Mountains is significantly different from other known species of the same genus. Therefore, we describe it here as a new species.

#### 3.2.2. *Gekko* (*Japonigekko*) *kaiyai* **sp. nov.** Zhang, Wu, and Zhang

http://zoobank.org/urn:LSID:zoobank.org:act:urn:lsid:zoobank.org:pub:E835D884-A34A-4A5D-84D3-CDE2006346AC (accessed on 11 November 2023).

**Figure 4:**
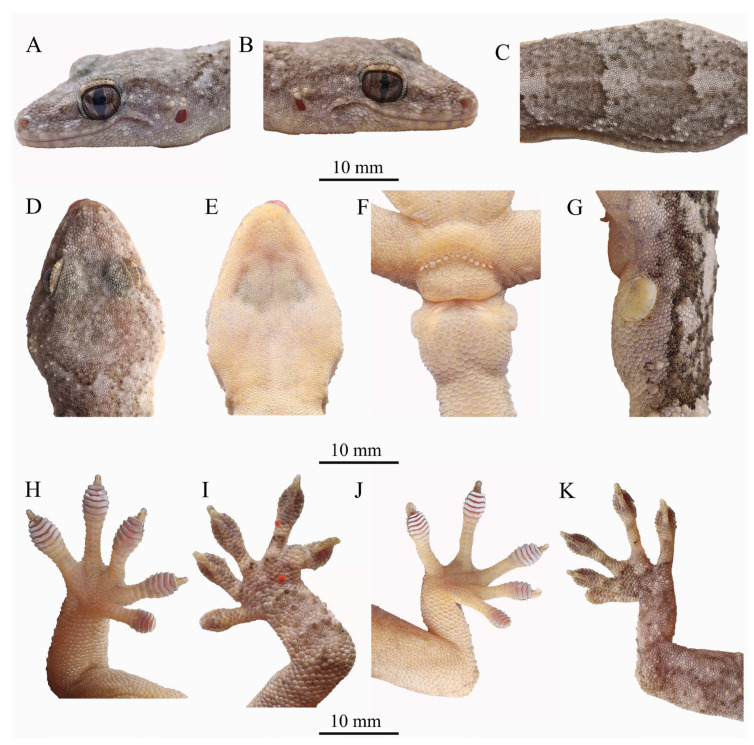
Holotype AHUXXBH07 of *Gekko kaiyai* **sp. nov.** in real life: (**A**), left lateral view of head, (**B**), right lateral view of head, (**C**), dorsal view of middle body, (**D**), ventral view, (**E**), dorsal view of head, (**E**), ventral view of head, (**F**), ventral view of pre-cloacal region, showing six pre-cloacal pores, (**G**), lateral view of basal tail, (**H**), dorsal view of hand, (**I**), ventral view of hand, (**J**), dorsal view of foot, (**K**), ventral view of foot.

**Holotype.** AHUXXBH007 (Figure 4), adult male, collected by C.W. Zhang, A.F. Wu and L.R. Wang in Liankang Mountain National Nature Reserve (31.6157° N, 114.8340° E; elevation 275 m a. s. l.), Xin County, Xinyang City, Henan Province, China on 14 July 2022.

**Paratype.** Sixteen specimens (two subadults, four adult females and 10 adult male), collected from the three locations by C.W. Zhang, A.F. Wu, S.L. Yu, X.N. Li, and L.R. Wang. Five specimens AHULXBH001-005 collected on 19 August 2022 from the Dongzhai National Nature Reserve, Luo County, Xinyang City, Henan Province, China. Nine adult and one subadult specimen of AHUXX001-006 and 008-011, respectively, were collected on the same day and in the same location as that of the holotype. One subadult specimen of AHUJGT001 was collected on 19 August 2022, from the Henan Dabie Mountains National Nature Reserve, Shangcheng County, Xinyang City.

**Etymology.** The specific, *Gekko kaiyai* **sp. nov.**, a Latinized adjective, was named after Professor Kaiya Zhou of the School of Life Sciences, Nanjing Normal University, China, who has made great contributions to the classification of the Gekkonidae family species, especially *Gekko hokouensis*. The suggested common English name is “Dabie Mountains Gecko” and the Chinese name is “Dà Bié Shān Bì Hŭ”, both of which indicate the location from where the new species was collected (Figure 1).

**Diagnosis.** *Gekko kaiyai* **sp. nov.** is distinguished from the subgenus *Japonicgekko* by its morphological characteristics: (1) medium body size (SVL 56.98–64.99 mm, n = 4, females; 50.03–61.56 mm, n = 11, males); (2) nostrils in contact with rostral scale; (3) tubercles on the dorsal, hindlimb and lower forelimb, but the upper forelimb smooth without tubercles; (4) interorbital scales between the anterior corners of the eyes 22–33; (5) ventral scales between mental and cloacal slit 157–209; (6) midbody scale rows 99–121; (7) ventral scale rows 30–43; (8) sub-digital lamellae on first fingers 7–9, on fourth fingers 8–13, on first toes 7–9, on fourth toes 7–11; (9) free of webbing in the fingers and toes; (10) 9–12 pre-cloacal pores in males and absent in females; (11) post-cloacal unilateral tubercles 1 (few 2); (12) and dorsum greyish white to dark brown, with 6–7 brown markings between the nape and sacrum.

**Description of holotype.** Adult male (AHUXXBH007, Figure 4) total length was 116.82 mm (SVL 53.3 mm, TaL 63.52 mm); tail length slightly longer than snout vent (SVL/TaL ratio, 83.9%); head relatively long (HL/SVL ratio, 26.5%) and distinctly longer than the width (HW/HL ratio, 83.8%), not markedly depressed (HH/HL ratio, 47.2%), distinct from neck; rostral scale approximate rectangular, wider than height (RW/RH ratio, 139.3%) and longer than mental (RW/MW ratio, 118.9%) in contact with the first supra-labial and supra-nasal on each side; nostril suborbicular, each surrounded by rostral scale, first supra-labial, supra-nasal and two enlarged post-nasal scales; naso-rostrals enlarged, in contact with each other; supra-nasal slightly smaller than post-nasal; two enlarged supra-nasals separated by a single oval inter-nasal; snout length moderate (SE/HL ratio, 37.7%), larger than eye diameter (SE/ED ratio,129.5%); snout region medial with flat from interorbital region to rostral scale; lateral snout scales oval, somewhat convex, twice larger than those in the interorbital region; preorbitals 13/13 (L/R); interorbitals 30; eye relatively large (ED/HL ratio, 24.7%); pupil vertical with crenulated margin; ear opening oval, obliquely oriented, smaller than eye (TD/ED ratio, 33.2%); mental pentagons, width shorter than length (MW/ML ratio, 51.2%); two enlarged post-mental scales, hexagonal, length longer than width; post-mentals in contact with mental and first infra-labials anteriorly and five gular scales posteriorly; supra-labial to midpoint of orbit 11/12; and infra-labial to midpoint of orbit 11/12.

Body slender, trunk relatively long (AG/SVL ratio, 50.1%); dorsal scales on body granular, smooth, round or oval, granular, juxtaposed; dorsal scales on body, nearly homogeneous, intermixed with distinctly enlarged tubercles (smooth, round to oval, 3–4 times the size of adjacent scales), surrounded by approximately 10 granular scales at the midbody; tubercles in 18 regular rows at the midbody; many tubercles also present in the temporal and occipital regions, as well as the limbs and dorsal surface of the tail; only the upper forelimb is smooth without tubercles; ventrolateral fold weakly developed, with many tubercles; ventral scales larger than the dorsals, smooth, hexagonal, and imbricate, much larger in the pre-cloacal region; 38 rows of ventral scales across the midbody; 12 pre-cloacal pores in the continuous series, each borne from a slightly enlarged scale. Base of the tail distinctly swollen, with one post-cloacal tubercle scales on each lateral side.

Forelimbs and hindlimbs well developed, moderately long, slender, tubercles on fore and hind limbs are present but absent from the upper forelimb; forelimb and tibia moderately long; digits moderately dilated, and all clawed except for digit I and toe I; free of webbing in the fingers and toes; sub-digital lamellae, unnotched, undivided: 8-8-8-8-7 (left manus), 6-6-7-7-7 (right manus), 9-9-8-7-7 (left pes), and 7-7-7-7-8 (right pes). Relative length of fingers: IV > III > V > II > I; relative length of toes: IV > III > V > II > I.

**Figure 5:**
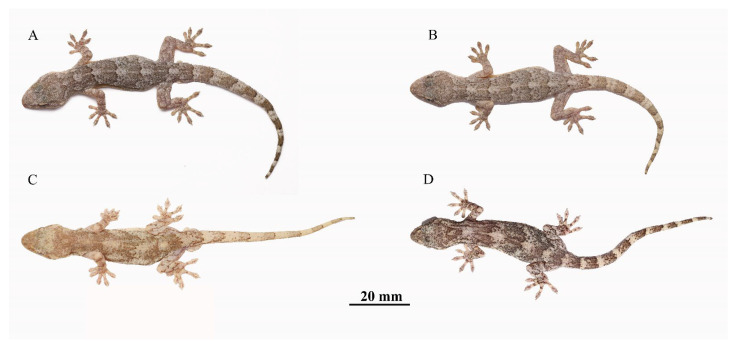
*Gekko kaiyai* **sp. nov.** and *G. hokouensis* dorsal view in life. (**A**), Holotype, AHUXXBH007, male. (**B**), Paratype, AHUXXBH010, female. (**C**), *G. hokouensis*, AHUWFSBH003, male, from Lujiang county, Anhui Province. (**D**), *G. hokouensis*, AHUQPBH003, female, from Jinzhai county, Anhui Province.

**Color of holotype in life.** (Figure 5) In life, the dorsal surface colors of the head and body were greyish brown, with six dark brown wide irregular patches from the neck to the swollen section of the tail. Each patch diminished gradually to the sides of the body and was outlined by a dark brown border. A light grayish tubercle stripe, bordered above with dark brown, extended from the posterior corner of the eye, passing above the ear opening to the occiput. Ventral surfaces of the head, belly, and limbs were light yellow with sparse small black spots. Ground color of the tail was brown, with nine irregular white stripes, each outlined by an inconspicuous dark brown wavy border. Limbs were light grey with greyish brown bars; dorsal surfaces of the limbs were flesh red.

**Figure 6:**
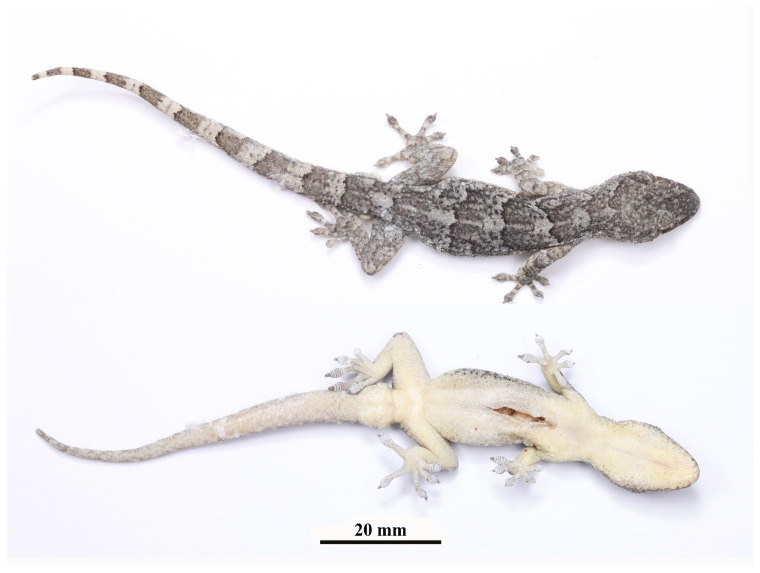
Holotype (AHUXXBH007, male) of *Gekko kaiyai* **sp. nov.** in preservative. Above, dorsal view. Below, ventral view.

**Color of holotype in preservative.** (Figure 6) In the preservative, the recently preserved specimens resemble those of the living body, and the pale grey coloration on the dorsal surface of the body and limbs becomes darker with increasing storage time. The light yellow body color fades to white with more prominent black spots.

**Variation.** Measurements and scale counts of the type series specimens are given in Table S4. Ground color on the dorsal surfaces of the head, body, and tail differ between individuals from yellowish grey to blackish grey in the wild, but the bodies of most individuals became darker after capture.

**Figure 7:**
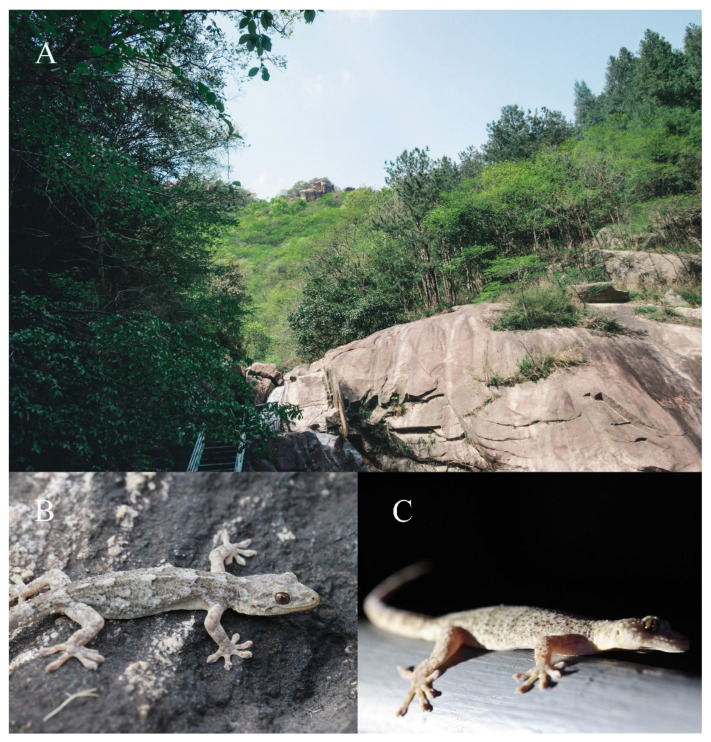
Habitat of *Gekko kaiyai* **sp. nov.** (**A**), Liankang Mountain National Nature Reserve, Xin County, Henan Province (photo by Kui Yang), (**B**), microhabitats, one gecko hides on a stone, (**C**), microhabitats, one gecko on the fence.

**Distribution and ecology.** *Gekko kaiyai* **sp. nov.** is only known from its type-locality, Liankang Mountains National Nature Reserve, Dongzhai National Nature Reserve, and Henan Dabie Mountains National Nature Reserve in the Dabie Mountains, Henan, China (Figure 1). The new species is nocturnal, inhabits scenic fences and rocky cliffs, and is found on the walls of buildings in the countryside of low mountain and hilly areas (Figure 7). Similar habitats were identified in the survey in other areas of the Dabie Mountains. It is thus reasonable to speculate that there may be distribution in other adjacent areas of the Dabie Mountains, Anhui Province.

#### 3.2.3. Comparison

**Figure 8:**
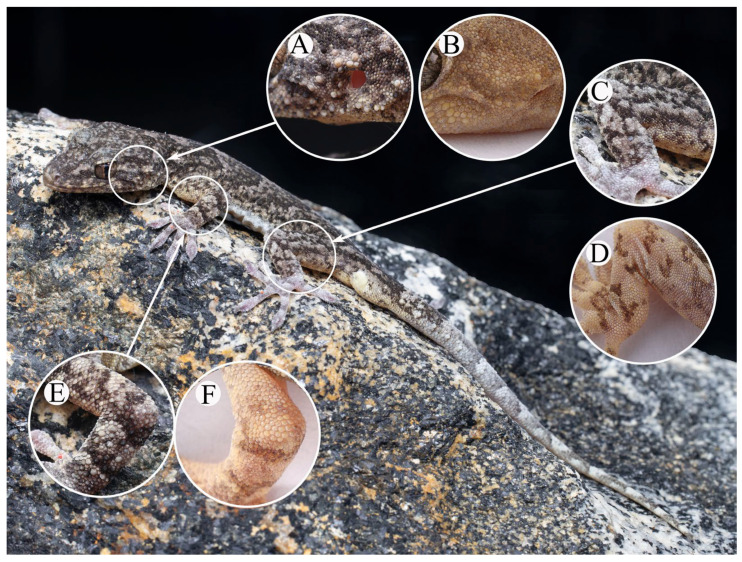
The difference between Holotype (AHUXXBH07, male) of *Gekko kaiyai* **sp. nov.** and *G. hokouensis* in real life. (**A**,**B**) lateral view of head with more tubercles between eyes and ears. (**C**,**D**) hindlimbs. (**E**,**F**) forelimbs.

**Figure 9:**
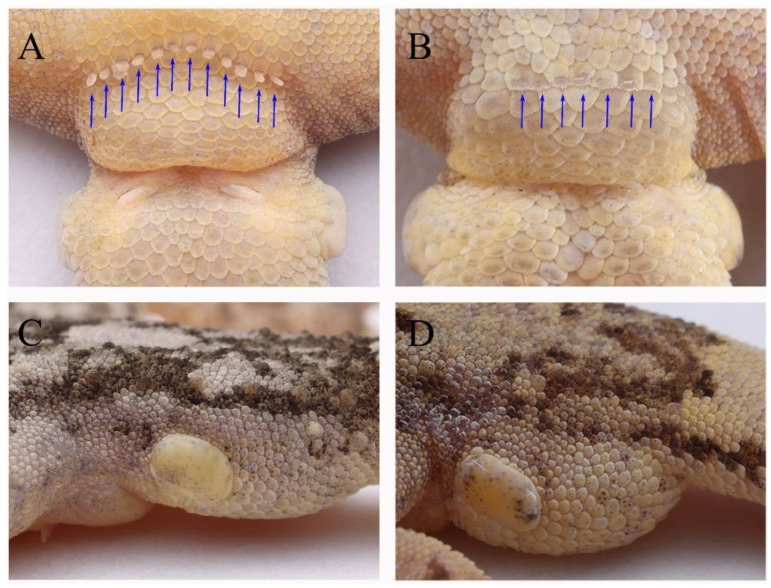
Male sexual character and basal tail of *Gekko kaiyai* **sp. nov.** (Holotype AHULKSBH007, male) and *G. hokouensis* (AHUHFBH01, male). (**A**,**C**) *G. kaiyai* **sp. nov.**; (**B**,**D**) *G. hokouensis*. (**A**,**B**) pre-cloacal region; (**C**,**D**) lateral view of basal tail.

Two species of the subgenus *Japonicgekko* have been reported in the Dabie Mountains (*Gekko hokouensis* and *G. japonicus*). However, the new species is the sister species of *G. hokouensis*, they are morphologically similar, but can be distinguished from *G. hokouensis* by the following morphological characteristics: fewer sub-digital lamellae under the fourth toe (7–11 vs. 15–18), tubercles on the limbs (present vs. absent), more pre-cloacal pores in the males (mostly 9–12 vs. 5–9), and more tubercles between the eyes and ears (Table 4; Figure 5, Figure 8 and Figure 9). The new species can be easily distinguished from *G. japonicus* as it has fewer scale rows at the midbody (99–121 vs. 130–144), fewer sub-digital lamellae under the first and fourth toes (first toe, 8–9 vs. 10–12; fourth toe, 7–11 vs. 14–16), tubercles on the thigh (present vs. absent), and fewer dorsal midline tubercle scales (sparse vs. dense).

*Gekko kaiyai* **sp. nov.** clearly differs from other non-sympatric species of the subgenus *Japonicgekko* by a unique suite of characteristics. The new species differ from the members of the subgenus *Japonicgekko* as follows (Table 4).

*Gekko kaiyai* **sp. nov.** can be easily distinguished from *G. aaronbaueri* as it has fewer supra-labials (9–12 vs. 13–14), fewer interorbital scales (22–33 vs. 34–37), dorsal tubercle rows at the midbody (10–16 vs. absent), fewer sub-digital lamellae under the first and fourth toes (first toe, 8–9 vs. 14–17; fourth toe, 7–11 vs. 14–16), tubercles on the limbs and dorsal surface of the tail (present vs. absent), and males have more pre-cloacal pores (9–12 vs. 3–4).

*Gekko kaiyai* **sp. nov.** can now be easily distinguished from *G. adleri* as it has fewer scale rows at the midbody (99–121 vs. 123–144), fewer sub-digital lamellae under the first and fourth toes (first toe, 8–9 vs. 11–14; fourth toe, 7–11 vs. 11–15), free of webbing (vs. present), tubercles on the forelimbs (present vs. absent), and males have fewer pre-cloacal pores (9–12 vs. 17–21).

*Gekko kaiyai* **sp. nov.** can be easily distinguished from *G. auriverrucosus* as it has a nostril touching rostral scale (vs. not touching) and enlarged post-mentals (2 post-mentals enlarged vs. 3 post-mentals enlarged).

*Gekko kaiyai* **sp. nov.** can be easily distinguished from *G. bonkowskii* as it has inter-nasals (1 vs. absent), more dorsal tubercle rows at the midbody (10–16 vs. absent), fewer sub-digital lamellae under the first and fourth toes (first toe, 8–9 vs. 11–13; fourth toe, 7–11 vs. 15), free of webbing (vs. present), tubercles on the limbs and tail (present vs. absent), and more pre-cloacal pores in the males (9–12 vs. 6).

*Gekko kaiyai* **sp. nov.** can be easily distinguished from *G. canhi* as it has fewer supra-labials (9–12 vs. 14), fewer interorbital scales (22–33 vs. 49–50), fewer scale rows at the midbody (99–121 vs. 205–227), fewer ventral scale rows at the midbody (30–43 vs. 49–51), fewer sub-digital lamellae under the first and fourth toes (first toe, 8–9 vs. 13–16; fourth toe, 7–11 vs. 14–17), tubercles on the forelimbs and tail (present vs. absent), and males have more pre-cloacal pores (9–12 vs. 5).

*Gekko kaiyai* **sp. nov.** can be easily distinguished from *G. chinensis* as it has fewer interorbital scales (22–33 vs. 49–50), more dorsal tubercle rows at midbody (10–16 vs. 10), is free of webbing (vs. present), has tubercles on the forelimbs (present vs. absent), and males have fewer pre-cloacal pores (9–12 vs. 17–21).

*Gekko kaiyai* **sp. nov.** can be easily distinguished from *G. cib* as it has dorsal tubercle rows at the midbody (10–16 vs. absent), fewer scale rows at the midbody (99–121 vs. 128–149), free of webbing (vs. present), and tubercles on the limbs and tail (present vs. absent).

*Gekko kaiyai* **sp. nov.** can be easily distinguished from *G. jinjiangensis* as males have more pre-cloacal pores (9–12 vs. 4–5).

*Gekko kaiyai* **sp. nov.** can be easily distinguished from *G. khunkhamensis* as it has inter-nasals (1 vs. absent), fewer scale rows at the midbody (99–121 vs. 127–138), fewer sub-digital lamellae under the first and fourth toes (first toe, 8–9 vs. 13–14; fourth toe, 7–11 vs. 14–15), is free of webbing (vs. present), has tubercles on the limbs and dorsal surface of the tail (present vs. absent), and males have more pre-cloacal pores (9–12 vs. absent).

*Gekko kaiyai* **sp. nov.** can be easily distinguished from *G. kwangsiensis* as it has fewer scale rows at the midbody (99–121 vs. 143–156), fewer sub-digital lamellae under the first and fourth toes (first toe, 8–9 vs. 11–13; fourth toe, 7–11 vs. 13–18), is free of webbing (vs. present), and has tubercles on the limbs (present vs. absent).

*Gekko kaiyai* **sp. nov.** can be easily distinguished from *G. lauhachindai* as it has a nostril touching rostral scale (vs. not touching), more inter-nasal–14; fourth toe, 7–11 vs. 13–15), is free of webbing (vs. present), has tubercles on the hind limbs s (1 vs. absent), fewer sub-digital lamellae under the first and fourth toes (first toe, 8–9 vs. 12 and tail (present vs. absent), and males have more pre-cloacal pores (9–12 vs. absent).

*Gekko kaiyai* **sp. nov.** can be easily distinguished from *G. liboensis* as it has inter-nasals (1 vs. absent), fewer interorbital scales (22–33 vs. 40), enlarged post-mental scales (vs. not enlarged), and tubercles on limbs (present vs. absent).

*Gekko kaiyai* **sp. nov.** differs from *G. melli* by having fewer interorbital scales (22–33 vs. 34–40), enlarged post-mental scales (vs. absent), dorsal tubercle rows at the midbody (11–18 vs. absent), fewer scales in a line from the mental to the front of the cloacal slit (153–176 vs. 181–200), fewer scale rows around the midbody (99–121 vs. 147–160), fewer sub-digital lamellae under the first toe and fourth toe (8–9 vs. 12–14), is free of webbing (vs. present), and has tubercles on the hind limbs and tail (present vs. absent).

*Gekko kaiyai* **sp. nov.** can be easily distinguished from *G. nadenensis* as it has more inter-nasals (1 vs. absent), dorsal tubercle rows at the midbody (10–16 vs. absent), fewer sub-digital lamellae under the first and fourth toes (first toe, 8–9 vs. 13–15; fourth toe, 7–11 vs. 14–16), free of webbing (vs. present), tubercles on the limbs and tail (present vs. absent), and more pre-cloacal pores in the males (9–12 vs. 6)

*Gekko kaiyai* **sp. nov.** differs from *G. palmatus* as it has fewer sub-digital lamellae under the first toe (8–9 vs. 13–15), is free of webbing (vs. present), has tubercles on the limbs (present vs. absent), and males have fewer pre-cloacal pores (9–12 vs. 23–30).

*Gekko kaiyai* **sp. nov.** differs from *G. scabridus* as it has tubercles on its tail (small vs. enlarged), as well as post-cloacal unilateral tubercles (1–2 vs. 2–3).

*Gekko kaiyai* **sp. nov.** can be easily distinguished from *G. scientiadventura* as it has more inter-nasals (1 vs. absent), fewer interorbital scales (22–33 vs. 41–51), dorsal tubercle rows at the midbody (10–16 vs. absent), more scales in a line from the mental to the front of the cloacal slit (157–209 vs. 118–140), fewer scale rows around the midbody (99–121 vs. 139–143), fewer sub-digital lamellae under the first and fourth toe (first toe, 8–9 vs. 12–15; fourth toe, 7–11 vs. 14–17), free of webbing (vs. present), tubercles on the hind limbs and tail (present vs. absent), and males have more pre-cloacal pores (9–12 vs. 5–8).

*Gekko kaiyai* **sp. nov.** can be easily distinguished from *G. sengchanthavongi* as they have more inter-nasals (1 vs. absent), dorsal tubercle rows at the midbody (10–16 vs. absent), fewer sub-digital lamellae under the first and fourth toe (first toe, 8–9 vs. 11–14; fourth toe, 7–11 vs. 13–17), free of webbing (vs. present), tubercles on the limbs and tail (present vs. absent), and males have more pre-cloacal pores (9–12 vs. 4–5).

*Gekko kaiyai* **sp. nov.** can be easily distinguished from *G. shibatai* as they have fewer interorbital scales (22–33 vs. 37–52), enlarged post-mental scales (present vs. absent), tubercles on the hind limbs (present vs. absent), tubercles on the limbs (present vs. absent), and males have more pre-cloacal pores (9–12 vs. 0–3).

*Gekko kaiyai* **sp. nov.** can be easily distinguished from *G. similignum* as they have fewer interorbital scales (22–33 vs. 46–48), enlarged post-mental scales (present vs. absent), fewer scale rows around the midbody (99–121 vs. 144–153), fewer sub-digital lamellae under the first and fourth toes (first toe, 8–9 vs. 11–13; fourth toe, 7–11 vs. 12–14), are free of webbing (vs. present), have tubercles on the hind limbs (present vs. absent), and fewer pre-cloacal pores in males (9–12 vs. 17).

*Gekko kaiyai* **sp. nov.** can be easily distinguished from *G. subpalmatus* as they have enlarged post-mental scales (present vs. absent), dorsal tubercle rows at the midbody (10–16 vs. absent), more lamellae under the fourth toe (11–18 vs. absent), fewer ventral scale rows at the midbody (30–43 vs. 48), free of webbing (vs. present), and tubercles on the hind limbs and tail (present vs. absent).

*Gekko kaiyai* **sp. nov.** can be easily distinguished from *G. swinhonis* as they have enlarged post-mental scales (present vs. absent), and more dorsal tubercle rows at the midbody (11–16 vs. 6–8).

*Gekko kaiyai* **sp. nov.** can be easily distinguished from *G. taibaiensis* as they have more lamellae under the first toe (8–9 vs. 6–7), tubercles on hind limbs (present vs. absent), and more pre-cloacal pores in the males (9–12 vs. 0–3).

*Gekko kaiyai* **sp. nov.** can be easily distinguished from *G. tawaensis* by having fewer supra-labials (9–12 vs. 15), fewer inter-nasals (1 vs. 2), enlarged post-mental scales (present vs. absent), more dorsal tubercle rows at the midbody (10–16 vs. absent), fewer sub-digital lamellae under the first and fourth toes (first toe, 8–9 vs. 10; fourth toe, 7–11 vs. 12), tubercles on the hind limbs and tail (present vs. absent), and males have more pre-cloacal pores (9–12 vs. absent).

*Gekko kaiyai* **sp. nov.** can be easily distinguished from *G. thakhekensis* by having more inter-nasals (1 vs. absent), more dorsal tubercle rows at the midbody (11–18 vs. absent), fewer sub-digital lamellae under the first and fourth toes (first toe, 8–9 vs. 11–13; fourth toe, 7–11 vs. 14–15), free of webbing (vs. present), tubercles on the fore limbs and tail (present vs. absent), and more pre-cloacal pores (9–12 vs. 1–5).

*Gekko kaiyai* **sp. nov.** can be easily distinguished from *G. truongi* by having fewer supra-labials (9–12 vs. 13–15), fewer interorbital scales (22–33 vs. 45–48), more dorsal tubercle rows at the midbody (10–16 vs. absent), fewer scale rows at midbody (99–121 vs. 131–143), fewer sub-digital lamellae under the first toe and fourth toe (first toe, 8–9 vs. 11–13; fourth toe, 7–11 vs. 15–17), and tubercles on the fore limbs and tail (present vs. absent).

*Gekko kaiyai* **sp. nov.** can be easily distinguished from *G. vertebralis* by having fewer interorbital scales (22–33 vs. 35–50), enlarged post-mental scales (vs. not enlarged), tubercles on the limbs and tail (present vs. absent), and males have more pre-cloacal pores (9–12 vs. 0–1).

*Gekko kaiyai* **sp. nov.** can be easily distinguished from *G. vietnamensis* as they have fewer interorbital scales (22–33 vs. 38–46) and males have more pre-cloacal pores (9–12 vs. absent).

*Gekko kaiyai* **sp. nov.** can be easily distinguished from *G. wenxianensis* as they have more dorsal tubercle rows at the midbody (10–16 vs. 10), more lamellae under the first toe (8–9 vs. 6), and more pre-cloacal pores (9–12 vs. 6–8).

*Gekko kaiyai* **sp. nov.** can be easily distinguished from *G. yakuensis* as they have enlarged post-mental scales (vs. not enlarged), fewer sub-digital lamellae under the first and fourth toe (first toe, 8–9 vs. 10; fourth toe, 7–11 vs. 15), tubercles on the limbs (present vs. absent), more lamellae under the first toe (8–9 vs. 6), and more pre-cloacal pores (9–12 vs. 6–8).

## 4. Discussion

The discovery of *Gekko kaiyai* **sp. nov.** has brought the total number of known species in the genus *Gekko* to 87 and the number identified within China to 22 [1,2,3,4,5,6,46]. The definition of a species is one of the basic concerns in biological research and is thus the focus of substantial taxonomic and systematic research [47]. Incorporating multiple lines of evidence to reveal the taxonomic and evolutionary relationships among species is now considered a standardized method in evolutionary biology [48]. In the field investigation, we had previously mistakenly identified the new species as *G. swinhonis*, because two species share many common attributes, such as the number of supra-labials, infra-labials, ventral scale rows at midbody, and interorbital scales. In our study, the identification of *G. kaiyai* **sp. nov.** as a sister to *G. hokouensis* was surprising, as they differed greatly in their morphologies, such as tubercles on the limbs, number of pre-cloacal pores, and number of tubercles between the eyes and ears. Nevertheless, the phylogeny and morphological difference revealed distinct divergences for *G. kaiyai* **sp. nov.** when compared with *G. hokouensis* and *G. japonicus* in the Dabie Mountains.

This investigation shows that the *Gekko kaiyai* **sp. nov.** only occurs in the northwest region of the Dabie Mountains (junction of Anhui and Henan provinces), China, while *G. hokouensis* is distributed in the eastern region of the Dabie Mountains, and *G. japonicus* is distributed in the plains around the Dabie Mountains. The ecological niches of these three gecko species have clearly been differentiated in the Dabie Mountains, and *G. kaiyai* **sp. nov.** was found to be predominantly distributed on buildings, rocks, and trees in the low hills (currently, only know distributed in the northwest of the Dabie Mountains), whereas *G. hokouensis* was mainly distributed in houses and woodlands (distributed throughout the Dabie Mountains), and *G. japonicus* is mainly distributed in houses on the plains (plain areas around the Dabie Mountains). In addition, we found form our multiple observation that *G. kaiyai* **sp. nov.** had strong aggressive tendencies and its tail did not break easily. The detailed distribution range, population size, and feeding habits of these three species in the Dabie Mountains has not yet been elucidated, and further investigations will be required to enhance our understanding of the interspecific relationships and sympatric distributions of these three species of gecko.

## 5. Conclusions

We described a new species of the gecko, *Gekko kaiyai* **sp. nov.**, based on the analysis of phylogeny and morphology. The discovery of this new species has brought the total number of known species in the genus *Gekko* to 87 and the number identified within China to 22. *Gekko kaiyai* **sp. nov.** appears currently only known to be distributed in the northwest of the Dabie Mountains, and with the *G. hokouensis* and *G. japonicus* are sympatric. However, the detailed distribution range, population size, and feeding habits of these three species in the Dabie Mountains has not yet been elucidated, and further investigations will be required to enhance our understanding of the interspecific relationships and sympatric distributions of these three species of gecko.

## Figures and Tables

**Table 1:** Samples used in the molecular analyses, including their GenBank numbers., Voucher ID and locality. “N/A” means no available data.

ID	Species	Locality	Voucher ID	16S	CYTB	COI	Renference
1	*G. kaiyai* **sp. nov.**	China: Henan: Xinyang: Xinxian	AHUXXBH01	OQ780318	OQ743839	OQ788612	This study
2	*G. kaiyai* **sp. nov.**	China: Henan: Xinyang: Xinxian	AHUXXBH02	OQ780319	OQ743840	OQ788613	This study
3	*G. kaiyai* **sp. nov.**	China: Henan: Xinyang: Xinxian	AHUXXBH03	OQ780320	OQ743841	OQ788614	This study
4	*G. kaiyai* **sp. nov.**	China: Henan: Xinyang: Luoxian	AHULXBH01	OQ780321	OQ743842	OQ788615	This study
5	*G. kaiyai* **sp. nov.**	China: Henan: Xinyang: shangcheng	AHUJGTBH01	OQ780322	OQ743843	OQ788616	This study
6	*G. kaiyai* **sp. nov.**	China: Henan: Xinyang: Xinxian	AHUXXBH06	OR381680	OR394955	OR394958	This study
7	*G. kaiyai* **sp. nov.**	China: Henan: Xinyang: Xinxian	AHUXXBH07	OR381681	OR394956	OR394959	This study
8	*G. kaiyai* **sp. nov.**	China: Henan: Xinyang: Xinxian	AHUXXBH10	OR381682	OR394957	OR394960	This study
9	*G. adleri*	China: Guangxi: Daxin County	SYSr001184	MW451636	MW448266	N/A	Lyu et al., 2021 [6]
10	*G. adleri*	China: Guangxi: Ningming County	SYSr001193	MW451640	MW448270	N/A	Lyu et al., 2021 [6]
11	*G. auriverrucosus*	China: Shanxi: Yuncheng	YC1	N/A	EU417692	EU417716	Zhou et al., 2008 [2]
12	*G. auriverrucosus*	China: Shanxi: Yuncheng	YC4	N/A	EU417695	EU417719	Zhou et al., 2008 [2]
13	*G. cib*	China: Sichuan: Chengdu	CD1	N/A	EU417696	EU417713	Zhou et al., 2008 [2]
14	*G. cib*	China: Sichuan: Chengdu City	CIB116961	MW451623	MW448256	N/A	Lyu et al., 2021 [6]
15	*G. cib*	China: Sichuan: Chengdu City	SYSr000708	MW451629	MW448260	N/A	Lyu et al., 2021 [6]
16	*G. chinensis*	China: Guangdong: Neilingding Island	SYSr001157	MW451634	MW448264	N/A	Lyu et al., 2021 [6]
17	*G. chinensis*	China: Guangdong: Shenzhen City	SYSr001085	MW451632	MW448262	N/A	Lyu et al., 2021 [6]
18	*G. hokouensis*	China: Anhui: Hefei: Lujiang	AHUHFBH01	OQ780323	OQ743844	N/A	This study
19	*G. hokouensis*	China: Anhui: Liuan: Shucheng	AHUSCBH01	OQ780324	OQ743845	OQ788617	This study
20	*G. hokouensis*	China: Fujian: Mt. Wuyi	SYSr001290	MW451647	MW448277	N/A	Lyu et al., 2021 [6]
21	*G. hokouensis*	China: Fujian: Shaowu City	SYSr001277	MW451646	MW448276	N/A	Lyu et al., 2021 [6]
22	*G. hokouensis*	China: Hunan: Hengdong County	SYSr001781	MW451665	MW448295	N/A	Lyu et al., 2021 [6]
23	*G. hokouensis*	China: Hunan: Mt. Hengshan	SYSr001319	MW451650	MW448280	N/A	Lyu et al., 2021 [6]
24	*G. hokouensis*	China: Jiangxi: Mt. Meiling	SYSr001311	MW451648	MW448278	N/A	Lyu et al., 2021 [6]
25	*G. hokouensis*	China: Anhui: Jinzhai	JZ1	N/A	EU417689	EU417720	Zhou et al., 2008 [2]
26	*G. japonicus*	China: Anhui: Liuan: Huoshan	AHULABH11	N/A	OQ743846	OQ788618	This study
27	*G. japonicus*	China: Guangxi: Guanyang County	SYSr001540	MW451656	MW448286	N/A	Lyu et al., 2021 [6]
28	*G. japonicus*	China: jiangxi: Lushan	SYSr001317	MW451649	MW448279	N/A	Lyu et al., 2021 [6]
29	*G. japonicus*	China: Zhejiang: Zhoushan	ZS1	N/A	EU417683	EU417723	Zhou et al., 2008 [2]
30	*G. japonicus*	China: Zhejiang: Zhoushan	ZS4	N/A	EU417686	EU417726	Zhou et al., 2008 [2]
31	*G. kwangsiensis*	China: Guangxi: Wuming County	SYSr001194	MW451641	MW448271	N/A	Lyu et al., 2021 [6]
32	*G. kwangsiensis*	China: Guangxi: Wuming County	SYSr001195	MW451642	MW448272	N/A	Lyu et al., 2021 [6]
33	*G. melli*	China: Guangdong: Mt. Yinping	SYSr001146	MW451633	MW448263	N/A	Lyu et al., 2021 [6]
34	*G. melli*	China: Guangdong: Mt. Yinping	SYSr001050	MW451631	MW448261	N/A	Lyu et al., 2021 [6]
35	*G. palmatus*	China: Guangxi: Napo County	SYSr001185	MW451637	MW448267	N/A	Lyu et al., 2021 [6]
36	*G. palmatus*	China: Guangxi: Nonggang Nature Reserve	SYSr001192	MW451639	MW448269	N/A	Lyu et al., 2021 [6]
37	*G. scabridus*	China: Yunnan: Dali	YNDL01	N/A	N/A	HM802949	Yan et al., 2010 [10]
38	*G. scabridus*	China: Yunnan: Dali	YNDL08	N/A	N/A	HM802950	Yan et al., 2010 [10]
39	*G. similignum*	China: Haihan: Mt. Wuzhi	SYSr001597	MW451658	MW448288	N/A	Lyu et al., 2021 [6]
40	*G. similignum*	China: Haihan: Mt. Wuzhi	SYSr001598	MW451659	MW448289	N/A	Lyu et al., 2021 [6]
41	*G. subpalmatus*	China: Zhejiang: Zhoushan Island	SYSr001381	MW451653	MW448283	N/A	Lyu et al., 2021 [6]
42	*G. subpalmatus*	China: Zhejiang: Fenghua County	SYSr001762	MW451662	MW448292	N/A	Lyu et al., 2021 [6]
43	*G. swinhonis*	China: Hebei: Zunhua County	SYS r001814	MW451666	MW448296	N/A	Lyu et al., 2021 [6]
44	*G. swinhonis*	China: Hebei: Zunhua County	SYS r001815	MW451667	MW448297	N/A	Lyu et al., 2021 [6]
45	*G. wenxianensis*	China: Gansu: Wenxian	WX1	N/A	EU417677	EU417703	Zhou et al., 2008 [2]
46	*G. wenxianensis*	China: Gansu: Wenxian	WX6	N/A	EU417682	EU417708	Zhou et al., 2008 [2]
	outgoup						
47	*G.* (*G.*) *gecko*	China: Guangxi: Nanning City	N/A	AY282753	AY282753	AY282753	Zhou et al., 2006 [12]

**Table 2:** Morphological comparison of *Gekko kaiyai* **sp. nov.** and *G. hokouensis*. All units in mm. *p*-values < 0.05 significance. Morphometric characters are explained in the methods section.

	Measurements									
	Male				Female				Male	Female
	*G. kaiyai* sp. nov. (n = 11)		*G. hokouensis* (n = 11)		*G. kaiyai* sp. nov. (n = 4)		*G. hokouensis* (n = 14)		*G. kaiyai* sp. nov. vs. *G. hokouensis*	*G. kaiyai* sp. nov. vs. *G. hokouensis*
	Range	Mean ± SE	Range	Mean ± SE	Range	Mean ± SE	Range	Mean ± SE		
SVL	50.03–61.56	57.97 ± 3.91	48.17–62.6	56 ± 497	56.98–64.99	62.3 ± 3.13	50.26–63.11	55.77 ± 4.83	0.326	0.0239 *
AG	22.96–30.95	26.91 ± 2.32	17.89–27.17	23.28 ± 2.36	27.68–34.24	30.57 ± 2.63	27.68–34.24	25.01 ± 2.98	0.0006 ***	0.0136 *
HL	13.34–15.73	14.83 ± 0.73	13.01–16.33	14.55 ± 0.93	15.71–16.14	15.94 ± 0.86	15.71–16.14	14.44 ± 1.03	0.94	0.00194 **
HW	11.39–13.48	12.63 ± 0.60	10.38–12.53	11.52 ± 0.71	12.96–13.77	13.26 ± 0.84	12.96–13.77	11.26 ± 1.22	0.0000 ***	0.0006 ***
HH	4.30–6.71	5.34 ± 0.56	4.48–8.05	6.31 ± 0.87	5.05–5.46	5.3 ± 0.61	5.05–5.46	6.14 ± 0.69	0.0005 ***	0.0164 *
SE	4.05–4.98	4.59 ± 0.25	4.06–5.81	4.93 ± 0.59	4.49–5.15	4.79 ± 0.44	4.49–5.15	4.63 ± 0.42	0.0552	0.783
ED	2.9–3.9	3.41 ± 0.25	2.47–3.18	2.87 ± 0.22	3.11–3.27	3.21 ± 0.40	3.11–3.27	2.95 ± 0.28	0.0000 ***	0.352
TD	0.97–1.56	1.22 ± 0.19	0.51–0.97	0.79 ± 0.15	1.31–1.68	1.53 ± 0.29	1.31–1.68	0.99 ± 0.34	0.0001 ***	0.0074 **
EE	3.57–4.88	4.37 ± 0.37	3.91–5.05	4.45 ± 0.36	4.61–4.98	4.78 ± 0.37	4.61–4.98	4.5 ± 0.57	0.204	0.882
RW	1.95–2.97	2.59 ± 0.32	1.57–2.23	1.95 ± 0.67	2.64–2.94	2.84 ± 0.43	2.64–2.94	1.98 ± 0.37	0.244	0.0000 ***
RH	0.93–1.4	1.13 ± 0.13	0.61–5.35	2.36 ± 0.67	0.97–1.35	1.18 ± 0.20	0.97–1.35	1.07 ± 0.22	0.1	0.361
ML	1.52–2.13	1.74 ± 0.17	1.53–1.92	1.78 ± 0.12	1.69–1.89	1.78 ± 0.15	1.69–1.89	1.85 ± 0.24	0.295	0.401
MW	0.66–0.99	0.85 ± 0.08	0.78–1.24	1.02 ± 0.13	0.93–1.1	1.03 ± 0.07	0.93–1.1	0.95 ± 0.19	0.0011 **	0.26

Note: * *p*-values < 0.05; ** *p*-values < 0.01; *** *p*-values < 0.001.

**Table 3:** Variable loadings with the first four principal component, with morphometric characters corrected.

Morphometric Characteristics	Female				Male			
	PC1	PC2	PC3	PC4	PC1	PC2	PC3	PC4
SVL	−0.2752	0.1692	0.3835	−0.0212	−0.1268	−0.1214	0.0492	0.9830
AG	−0.2496	0.3650	0.1457	−0.4916	−0.3759	−0.0181	0.2899	−0.0633
HL	−0.4581	−0.2206	0.0884	0.1963	0.1405	−0.3404	−0.7896	0.0224
HW	−0.0341	−0.1749	0.5379	−0.1723	−0.3297	−0.3630	−0.1878	−0.0832
HH	0.1627	−0.5465	−0.1819	−0.3927	0.4034	−0.0260	0.1856	0.0264
ED	−0.2986	0.2448	−0.5670	0.2064	−0.3050	−0.4042	−0.0188	−0.0976
TD	−0.4409	0.1503	−0.1211	0.2323	−0.2462	−0.5201	0.3002	−0.1048
EE	−0.1381	−0.5411	0.1123	0.4397	0.3066	−0.3896	0.1596	−0.0149
RH	−0.2892	−0.2212	−0.2958	−0.4689	0.3413	−0.3216	0.3247	−0.0037
MW	−0.3667	−0.1897	−0.2554	−0.1599	0.4329	−0.2105	−0.0054	0.0214
Eigenvalues	3.3832	1.8118	1.4060	0.9876	3.9305	2.0704	0.9878	0.9353
Percentage of total variance	34.8323	18.1183	14.0603	9.8761	39.3049	20.7039	9.8781	9.3625
Cumulative percentage	34.8323	52.9506	67.0109	76.8870	39.3049	60.0088	69.8869	79.2395

**Table 4:** Morphological comparisons among the species of the subgenera *Japonigekko*. “–” means data unavailable; Black bold fonts represent difference with the new species.

Characters	MaxSVL	SPL	IFL	N to R	I	IO	PM	DTR	SMC	SR	V	LT1	LT4	Web	Fore Tubercles	Hind Tubercles	Tail Tubercles	PP
*G. kaiyai* **sp. nov.**	64.99	9–12	9–13	1	1–1	22–33	1	11–18	157–209	99–121	30–43	8–9	7–11	0	1	1	1	9–12
*G. aaronbaueri*	80	**13–14**	10–11	1	0–1	**34–37**	1	**0–0**	–	98–104	39–43	**14–17**	**14–16**	–	**0**	**0**	**0**	**3–4**
*G. adleri*	75.3	10–15	9–13	1	1–1	27–36	1	7–11	168–190	**123–144**	35–44	**11–14**	**11–15**	**1**	**0**	1	1	**17–21**
*G. auriverrucosus*	69	9–11	9–11	**0**	0–1	25–25	**0**	16–20	–	–	–	6–8	6–8	0	1	1	1	8–11
*G. bonkowskii*	69.2	12–14	10–11	1	**0**	26–27	1	**0–0**	154–169	117–117	37–40	**11–13**	**15–15**	**1**	**0**	**0**	**0**	**6–6**
*G. canhi*	99.2	**14–14**	10–12	1	1–1	**49–50**	1	11–12	168–170	**205–227**	**49–51**	**13–16**	**14–17**	0	**0**	1	**0**	**5–5**
*G. chinensis*	72	10–14	9–13	1	1–1	**35–48**	1	**10–10**	156–167	118–140	37–39	8–10	9–12	**1**	**0**	1	1	**17–27**
*G. cib*	66.4	10–12	10–14	1	1–2	28–36	1	**0–0**	171–196	**128–149**	37–45	9–13	9–17	**1**	**0**	**0**	**0**	7–9
*G. hokouensi*	70	10–14	8–11	1	1–1	30–33	1	12–18	153–174	119–130	36–43	8–11	**15–18**	0	**0**	**0**	1	5–9
*G. japonicus*	74	9–13	8–13	1	0–1	32–35	1	9–14	169–188	**130–144**	39–44	**10–12**	**14–16**	0	1	1	1	6–9/4–8
*G. jinjiangensis*	61.6	7–10	6–9	1	0–1	20–24	1	12–16	146–169	111–149	31–47	8–11	11–15	0	1	1	1	**4–5**
*G. (J.)khunkhamensis*	75.2	10	9	–	**0**	31–32	–	–	181–185	**127–138**	42–45	**13–14**	**14–15**	**1**	**0**	**0**	**0**	**0–0**
*G. kwangsiensis*	69.7	10–12	11–13	1	0–1	29–31	1	9–11	185–208	**143–156**	41–45	**11–13**	**13–18**	**1**	**0**	**0**	1	9–11
*G. lauhachindai*	98	11–12	11	**0**	**0**	24	1	14	–	112–121	32	**12–14**	**13–15**	**1**	**0**	**0**	**0**	12–14
*G. liboensis*	85	12–12	11–11	1	**0**	**40–40**	**0**	**10–10**	–	–	–	8–8	9–9	0	**0**	**0**	–	–
*G. melli*	84.6	10–13	9–12	1	1–1	**34–40**	**0**	**0–0**	181–200	**147–160**	43–49	**10–12**	11–14	**1**	**0**	**0**	**0**	9–11
*G. nadenensis*	77.1	12–14	10–12	1	**0**	28–30	1	**0–0**	175–185	**123–140**	43–49	**13–15**	**14–16**	**1**	**0**	**0**	**0**	**6–6**
*G. palmatus*	79.7	11–15	9–13	1	0–3	27–36	1	4–12	160–191	116–147	36–47	**10–13**	10–16	**1**	**0**	**0**	1	**23–30**
*G. scabridus*	64	9–11	9–11	1	1–2	30–30	1	17–21	–	–	–	6–9	7–9	0	1	1	1	10–15
*G. scientiadventura*	73	12–14	9–13	1	**0**	**41–51**	1	**0–0**	**118–140**	**139–143**	38–48	**12–15**	**14–17**	**1**	**0**	**0**	**0**	**5–8**
*G. sengchanthavongi*	77.3	8–10	**6–7**	1	**0**	28–32	1	**0–0**	175–184	120–135	35–43	**11–14**	**13–17**	**1**	**0**	**0**	**0**	**4–5**
*G. shibatai*	70.9	10–13	10–14	1	0–1	**37–52**	**0**	5–14	–	114–134	–	–	9–16	0	**0**	**0**	1	**0–3**
*G. similignum*	58.9	12–14	11–11	1	1–1	**46–48**	**0**	11–11	–	**144–153**	–	**11–13**	**12–14**	**1**	**0**	**0**	1	**17–17**
*G. subpalmatus*	72	8–12	7–12	1	1–1	32–32	**0**	**0–0**	–	–	**48–48**	7–9	7–10	**1**	**0**	**0**	**0**	5–11
*G. swinhonis*	66	7–12	7–11	1	–	23–24	**0**	**6–8**	–	–	40–40	6–9	6–9	0	1	1	–	7–9
*G. taibaiensis*	69	9–10	8–10	1	–	28–28	–	–	–	–	–	**6–7**	7–8	–	–	–	–	**4–6**
*G. tawaensis*	71	**15–15**	13–13	1	**2–2**	–	**0**	**0–0**	–	–	–	**10–10**	**12–12**	0	**0**	**0**	**0**	**0–0**
*G. thakhekensis*	79.2	12–14	10–11	1	**0**	22–26	1	**0–0**	165–174	110–116	32–40	**11–13**	**14–15**	**1**	**0**	**0**	**0**	**1–5**
*G. truongi*	95.9	**13–15**	11–13	1	0–1	**45–48**	1	**0–0**	160–172	**131–143**	35–36	**11–13**	**15–17**	0	**0**	**0**	**0**	10–11
*G. vertebralis*	69.2	10–15	10–15	1	0–2	**35–50**	**0**	2–12	–	112–139	–	–	9–17	0	**0**	**0**	**0**	**0–1**
*G. vietnamensis*	91	11–12	10–11	–	–	**38–46**	–	–	–	–	–	–	–	0	–	–	–	**0–0**
*G. wenxianensis*	59	12–12	11–11	1	1–1	–	1	**10–10**	–	–	42–44	**6–6**	9–9	0	**0**	1	–	**6–8**
*G. yakuensis*	72	12–13	9–13	1	1–1	–	**0**	–	–	–	–	**10–10**	**15–15**	0	**0**	**0**	1	**6–8**

## Data Availability

The data presented in this study are available on request from the corresponding author.

## Supplementary Materials

The following supporting information can be downloaded at: https://www.mdpi.com/article/10.3390/ani13243796/s1, Table S1: Uncorrected pairwise distances within and between species of the subgenera Gekko based on 16s sequences, Table S2: Uncorrected pairwise distances within and between species of the subgenera Gekko based on CYTB sequences, Table S3: Uncorrected pairwise distances within and between species of the subgenera *Japonigekko* based on COI sequences, Table S4: The measurements (in mm) and morphological characters of *Gekko kaiyai* **sp. nov.** and measurements characters of *G. hokouensis*.
